# Combination therapy with afatinib and bevacizumab in an *EGFR*-mutated non-small cell lung cancer patient with acquired ERBB2 amplification: A case report

**DOI:** 10.1097/MD.0000000000024380

**Published:** 2021-02-26

**Authors:** Sixian Chen, Tianmin Xiang, Wei Lu, Shuiqiang Hong, Yuanyuan Li, Yuan Lu, Qiongyue Zhang, Yongfeng Chen, Suli Zhou, Gehui Wang, Zhenzhen Zhang, Yongguang Cai

**Affiliations:** a Medical Oncology Department V, Guangdong Nongken Central Hospital, Zhanjiang; b Singlera Genomics Inc., Shanghai, China.

**Keywords:** afatinib, bevacizumab, case report, *ERBB2* amplification, non-small cell lung cancer

## Abstract

**Introduction:**

Acquired resistance to reversible EGFR tyrosine kinase inhibitors remains a significant obstacle, and acquired *ERBB2* amplification is the most common “bypass” mechanism. For patients with sensitizing *EGFR* mutation who experience resistance via *ERBB2* amplification, no targeted drug has been demonstrated to be effective.

**Patient concerns:**

A 56-year-old female nonsmoker suffered from left leg paralysis and low back pain. Imaging examination revealed a mass in the anterior segment of the right upper lobe lung and possible multiple metastases in the right hilar, mediastinal lymph nodes, bone metastases, and soft tissue invasion.

**Diagnosis:**

Transbronchial lung biopsy revealed a moderately differentiated adenocarcinoma (cT4N2M1c, stage IV). An *EGFR* exon 19 deletion was identified using amplification refractory mutation system.

**Interventions:**

After the patient was treated with gefitinib initiation (250 mg/d) for 15 months, the tumor progressed with *ERBB2* amplification revealed by next-generation sequencing test. Then, the patient was started on afatinib (40 mg/d) plus bevacizumab (7.5 mg/kg every 3 weeks).

**Outcomes:**

The combination therapy of afatinib and bevacizumab in this patient was effective with some slight side effects. Computed tomography scans showed the tumor shrinkage and the pleural effusion disappeared in the right lung. The overall survival was 23.5 months.

**Conclusion:**

To date, there is no targeted therapy approved and demonstrated to be effective for non-small cell lung cancer patients with *EGFR* sensitizing mutations, and *ERBB2* amplification. The effectiveness of combination therapy with afatinib and bevacizumab may provide a new therapeutic option for these patients.

## 1 Introduction

Non-small cell lung cancer (NSCLC) patients with *EGFR*-sensitizing mutations are markedly responding to EGFR tyrosine kinase inhibitors (TKIs) therapy, such as gefitinib, erlotinib, and afatinib.^[1]^ Unfortunately, almost all the patients with this mutation will develop acquired resistance after the EGFR-TKI treatment.^[2]^ More than half of the cases resistant to the 1st and 2nd generation EGFR TKIs are due to a secondary T790M mutation, while some other cases with acquired resistance have been explored as “bypass” resistance mechanisms.^[3]^*ERBB2* amplification is one of the most common “bypass” mechanisms, which presents in 10% to 15% cases with acquired resistance of “bypass” mechanism.^[3]^ To date, there is no targeted drug demonstrated effective and approved by FDA for NSCLC patients with *EGFR* sensitizing mutations and *ERBB2* amplification. Here, we report 1 case who received a combination therapy of afatinib and bevacizumab and achieved promising clinical response.

## 2 Case report

**Figure 1:**
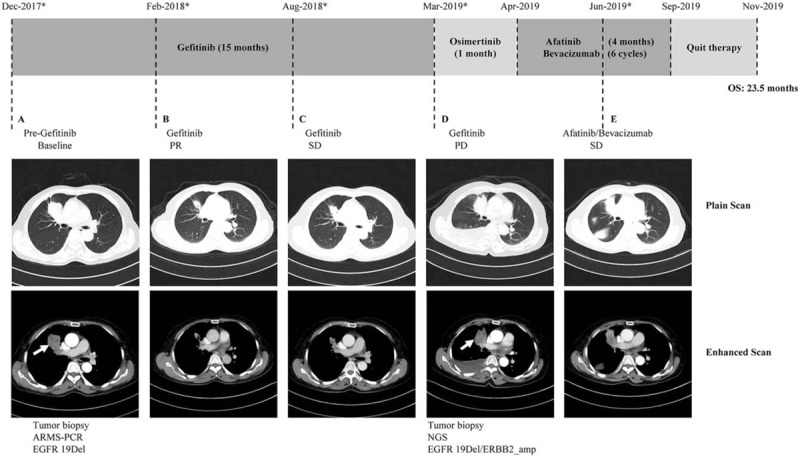
A clinical summary of the patient's treatment process. Upper panel shows the treatments for the patient with *EGFR*-mutated non-small cell lung cancer. Bottom panel shows computed tomography (CT) plain and enhanced scans of the patient's lung lesions before gefitinib therapy (A), partial response (PR) to gefitinib (B), stable disease (SD) on gefitinib (C), progressive disease (PD) to gefitinib (D), and SD on afatinib and bevacizumab (E). Genetic testing results of the biopsy tissues are shown below the corresponding CT images. The white arrow represents the position of sampling. ^∗^indicate the time points for response assessment.

**Figure 2:**
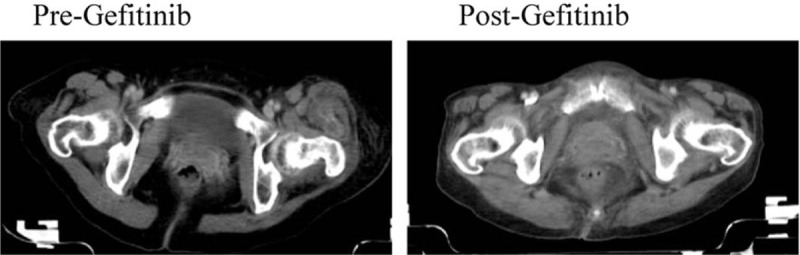
Pelvic CT images of the patient. Shrinkage of left femur metastases and decrease of soft tissue invasion after administration of gefitinib. CT = computed tomography.

A 56-year-old female nonsmoker came to hospital because of left leg paralysis and low back pain on December 1, 2017. Chest computed tomography (CT) scan revealed a mass in the anterior segment of the right upper lobe lung and possible multiple metastases in the right hilar and mediastinal lymph nodes (Fig. 1A). Cranial magnetic resonance imaging, pelvic CT, and whole-body bone scans revealed multiple bone metastases and soft tissue invasion. Biopsy tissue from right lung mass histologically showed a moderately differentiated adenocarcinoma (cT4N2M1c, stage IV) and amplification refractory mutation system identified an *EGFR* exon 19 deletion. The initial treatment regimen was started on 2 December 2017: intravenous infusion with 6 mg Sodium ibandronate dissolved in 0.9% Saline 500 mL, followed by Gamma Knife radiosurgery for left femur (3300 cGy/11f). Then, the patient was started on 1st generation EGFR-TKI gefitinib with a dose of 250 mg/d on 15 December 2017 and developed a partial response according to the CT plain and enhanced scans 2 months later (Fig. 1B). Her clinical symptoms including leg paralysis and low back pain were remarkably relieved without serious adverse events. Compared to pretreatment of gefitinib, CT images revealed shrinkage of left femur metastases and decrease of soft tissue invasion after administration of gefitinib (Fig. 2). In August 2018, CT plain and enhanced scans of the right lower lobe nodule (Fig. 1C) indicated stable disease (SD). However, after 15 months of gefitinib treatment, chest CT scans observed a sharp increase in the size of the right upper lobe mass, large right pleural effusion (PE) and nodular right pleural thickening, and magnetic resonance imaging showed metastases of the eighth thoracic vertebra. These situations demonstrated progressive disease in the lung cancer patient (Fig. 1D). Until this moment, the progression-free survival (PFS) was 15 months. The patient was then treated with a 3rd generation EGFR-TKI osimertinib (80 mg/d). Unfortunately, the patient developed cough associated with a lot of white sticky sputum and chest pain a month later, and no response to Osimertinib was observed. On April 18, 2019, the patient underwent right Closed Thoracic Drainage and received Nedaplatin (80 mgd1) plus Sapylin (8KE) by intrathoracic injection. Subsequently, cytology analysis identified adenocarcinoma cells from the PE, a re-biopsy turned out to be adenocarcinoma of the enlarged lesion (cT4N3M1c, stage IV), and immunohistochemistry findings showed that the tumor cells were positive for TTF-1 and NapsinA and negative for CK5/6. Tumor tissue collected by re-biopsy was analyzed by next generation sequencing with an OncoAim Lung cancer targeting gene detection kit (Singlera Genomics, Inc., Shanghai, China) and revealed an acquired *ERBB2* amplification (*ERBB2*_amp) in conjunction with *EGFR* 19 deletion mutation. After 1 cycle of Sodium ibandronate (6 mg), the patient was treated with afatinib at 40 mg/d plus bevacizumab at 7.5 mg/kg every 3 weeks. After 3 cycles of bevacizumab administration, CT scans showed the tumor lesions shrinkage and the PE disappeared (Fig. 1E), which was considered to be SD. The therapy caused some slight side effects, such as rash, diarrhea, hypertension, and inappetence. Regrettably, after 4 months of combination therapy, the patient quit all the therapy in September 2019, because of failure to pay the expensive medical bills. The overall survival (OS) was 23.5 months.

## 3 Discussion

Amplification of *ERBB2* is one of the mechanisms of acquired resistance to first-generation EGFR-TKI in *EGFR*-mutant NSCLC tumors that lack the *EGFR* T790M secondary mutation.^[4]^ In the current case, the amplification refractory mutation system test for the primary tumor demonstrated the presence of TKI sensitive mutation *EGFR* exon 19 deletion and it indeed showed good response to Gefitinib. When the resistance to EGFR-TKI occurred. Next-generation sequencing analyses for the recurred lesions revealed the existence of *ERBB2* amplification, which may reasonably explain this EGFR-TKI resistance

In the *EGFR*-mutated patient, activation of *ERBB2*, representing parallel or “bypass” signaling pathway that share downstream effectors with *EGFR*, has been demonstrated to make tumor cells less dependent on *EGFR* for activation of these downstream effectors, and thereby reduce the antitumor efficacy of EGFR-TKI.^[4]^ There are target drugs available for Her2 positive patients resulting from *ERBB2* amplification. However, to date, there is neither HER2-specific targeted therapies approved for *EGFR*-mutated patients who undergo resistance via *ERBB2* amplification, nor sufficient data to indicate whether these patients would benefit from HER2-targeted therapies. In the present case of metastatic lung adenocarcinoma with acquired EGFR-TKI resistance, the combination treatment with afatinib plus bevacizumab led to the stabilization of the disease.

Afatinib, a second-generation EGFR-TKI, is a selective and potent irreversible inhibitor of *EGFR* and ERBB-family.^[5]^ In a randomized phase III trial, afatinib treatment resulted in a significantly better response and prolonged PFS (11.1 months) compared with the standard doublet chemotherapy in advanced NSCLC patients harboring activating *EGFR* mutations (PFS = 6.9 months).^[6]^ Similarly, in another randomized phase III trial, median PFS was significantly longer in the afatinib group (11.0 months) than that in the gemcitabine and cisplatin group (5.6 months).^[7]^ The most common treatment-related adverse events were diarrhea, rash/acne, and stomatitis for afatinib in the above 2 clinical trials. Suzawa et al^[8]^ reported the antitumor effect of afatinib in vitro in NSCLC cells with *ERBB2* mutation or amplification, suggesting that afatinib was a therapeutic option as a HER2-targeted therapy for NSCLC patient with *ERBB2* alterations. Bevacizumab is an anti-angiogenic monoclonal antibody that blocks vascular endothelial growth factor.^[9]^ A phase 2 trial (JO25567) suggested that bevacizumab can postpone the resistance of tumor cells to TKI.^[10]^ Furthermore, a phase III study showed that bevacizumab plus EGFR-TKI significantly improved the PFS compared with EGFR-TKI alone in NSCLC patients with activating *EGFR* mutations (16.9 months vs 13.3 months), of which the most common grade 3 to 4 adverse event was rash, and a small number of patients had serious adverse events, like neutropenia and dysfunction.^[11]^ Another case revealed that the addition of bevacizumab (7.5 mg/kg once every 2 weeks) to afatinib (40 mg/d) treatment could overcome TKI resistance with no serious adverse events occurred throughout the treatment period.^[12]^ In a phase 2 trial, afatinib (30 mg/d) plus bevacizumab (15 mg/kg once every 3 weeks) therapy has demonstrated clinical efficacy and safety for patients with acquired resistance to EGFR TKIs.^[13]^ Grade 3 or higher adverse events (incidence ≥10%) included paronychia, hypertension, and proteinuria and no treatment-related deaths, interstitial lung disease, or bevacizumab-associated severe bleeding was observed. Based on the results of clinical trials mentioned above and individual tolerability, combination treatment with bevacizumab and afatinib was recommended and applied in the patient of our study; treatment with bevacizumab (7.5 mg/kg once every 3 weeks) combined with continued therapy of afatinib (40 mg/d). Furthermore, follow-up CT scans showed SD until the patient quit therapy, which suggested that the combination therapy of afatinib and bevacizumab could inhibit the tumor growth in EGFR-mutant patients with *ERBB2* amplification. No serious adverse events, such as neutropenia, pulmonary hemorrhage and uncontrolled hypertension, occurred throughout the treatment period. Although gene alteration in our report was different from that in these clinical trials mentioned above, it may be a good reference and attempt for *EGFR-*mutated NSCLC patients with *ERBB2* amplification adopting the combination therapy with afatinib plus bevacizumab. Compared to a phase 2 trial,^[13]^ it might be a better choice for 7.5 mg/kg bevacizumab in the advanced EGFR-mutated patients based on individual tolerability.

Several limitations of the present case should be acknowledged. First, the patient quit treatment because of her financial burden, implying that the true OS could have been underestimated. Second, molecular diagnosis has not been fully implemented in this hospital, which resulted in failure of evaluation whether there existed other *EGFR*-mutant cases with non-T790M-mediated resistance to gefitinib treatment. Based on the limitations in the current report, a further clinical trial with greater sample sizes need to be conducted to resolve these problems.

In conclusion, we have shown a promising combination therapy with afatinib and bevacizumab in an *EGFR*-mutated NSCLC patient with *ERBB2* amplification who had acquired resistance to gefitinib therapy. The OS was 23.5 months without obvious side effects and the efficacy evaluation was SD. However, further studies are required to confirm the finding.

## Author contributions

**Conceptualization:** Sixian Chen, Tianmin Xiang.

**Data curation:** Wei Lu, Qiongyue Zhang, Yongfeng Chen, Gehui Wang.

**Formal analysis:** Shuiqiang Hong, Suli Zhou.

**Funding acquisition:** Yongguang Cai.

**Methodology:** Yuanyuan Li, Yuan Lu.

**Writing – original draft:** Zhenzhen Zhang.
